# Synaptic loss in a mouse model of euthyroid Hashimoto’s thyroiditis: possible involvement of the microglia

**DOI:** 10.1186/s12868-022-00710-2

**Published:** 2022-04-25

**Authors:** Fen Wang, Yao-Jun Cai, Xiao Ma, Nan Wang, Zhang-Bi Wu, Yan Sun, Yong-xia Xu, Hao Yang, Tian-tian Liu, Qin Xia, Zhen Yu, De-Fa Zhu

**Affiliations:** 1 Department of Geriatric Endocrinology, The First Affiliated Hospital of Anhui Medical University, Hefei, 230032 China; 2 Department of Endocrinology, The First Affiliated Hospital of Nanchang University, Nanchang, 330006 China; 3 Department of Respiratoration, Wuhu Hospital of Traditional Chinese Medicine, Wuhu, 241000 China; 4 Department of Obstetrics and Gynaecology, The First Affiliated Hospital of Anhui Medical University, Hefei, 230032 China

**Keywords:** Hashimoto’s thyroiditis, Rodent model, Synaptic loss, Microglia, Phagocytosis

## Abstract

**Background:**

Hashimoto’s thyroiditis (HT) is an autoimmune illness that renders individuals vulnerable to neuropsychopathology even in the euthyroid state, the mechanisms involved remain unclear. We hypothesized that activated microglia might disrupt synapses, resulting in cognitive disturbance in the context of euthyroid HT, and designed the present study to test this hypothesis.

**Methods:**

Experimental HT model was induced by immunizing NOD mice with thyroglobulin and adjuvant twice. Morris Water Maze was measured to determine mice spatial learning and memory. The synaptic parameters such as the synaptic density, synaptic ultrastructure and synaptic-markers (SYN and PSD95) as well as the interactions of microglia with synapses were also determined.

**Results:**

HT mice had poorer performance in Morris Water Maze than controls. Concurrently, HT resulted in a significant reduction in synapse density and ultrastructure damage, along with decreased synaptic puncta visualized by immunostaining with synaptophysin and PSD-95. In parallel, frontal activated microglia in euthyroid HT mice showed increased engulfment of PSD95 and EM revealed that the synaptic structures were visible within the microglia. These functional alterations in microglia corresponded to structural increases in their attachment to neuronal perikarya and a reduction in presynaptic terminals covering the neurons.

**Conclusion:**

Our results provide initial evidence that HT can induce synaptic loss in the euthyroid state with deficits might be attributable to activated microglia, which may underlie the deleterious effects of HT on spatial learning and memory.

**Supplementary Information:**

The online version contains supplementary material available at 10.1186/s12868-022-00710-2.

## Background

Hashimoto’s thyroiditis (HT) is a prevalent autoimmune disorder that primarily impacts the thyroid and is characterized by intrathyroidal monocyte infiltration and elevated serum levels of autoantibodies specific for thyroid peroxidase (TPO-Ab) and thyroglobulin (Tg-Ab) [1]. The disease affects ~ 5% of the global population and is more common among females relative to males [2]. In the majority of cases (79.3%), normal thyroid function is observed at diagnosis before progression to hypothyroidism [3]. Emerging clinical data suggest an association of HT with neuropsychological impairment even in those with normal thyroid function [4–7]. Depression and anxiety are more prevalent among euthyroid HT patients relative to the general population [6]. Only in recent years, several clinical studies have reported an association between cognitive impairment and euthyroid HT [8, 9]. Leyhe et al. have observed attention deficits in euthyroid HT patients [10] that were correlated with a loss of frontal lobe gray matter density [11]. These findings strongly suggest central nervous system (CNS) involvement in HT, but the specific neural mechanisms underlying the regulation remain poorly understood.

Our previous study [12] found hippocampal-dependent learning and memory alterations in HT, which may attribute, at least partly, to astrocytes impairment. Another study reported by us [13] demonstrated that emotional behavior deficits in euthyroid HT model mice were related to microglial activation in the frontal lobe, which is an important brain region for the emotional processing and cognitive regulation. Microglia and CNS-resident macrophages are key mediators of brain development and homeostasis [14]. In response to local inflammation or brain damage, microglia can become reactive and can thereafter drive the pathogenesis of conditions such as brain trauma [15], Alzheimer’s disease [16], and depression [17]. Activated microglia secrete an array of neuroactive factors [18], including ATP, glutamate, free radicals, chemokines, and cytokines such as interleukin-1β (IL-1β) and tumor necrosis factor alpha (TNF-a) that potently regulate neuronal function [19].

Recent studies have explored the impact of microglial activation on neuronal synapses and associated cerebral homeostasis [20]. Specifically, microglia have been shown to shape synapse formation and to interact with synapses to regulate neuronal firing [21–23]. Activated microglia have been found to closely appose neurons in pathological settings [24–26], where they shape synaptic connections within neural circuits. These studies indicate that microglia have close interactions with synapses. Whether these reactive microglia are capable of promoting synaptic disruption in HT, however, remains to be determined. We hypothesized that activated microglia might disrupt synapses, resulting in cognitive disturbance in the context of euthyroid HT, and designed the present study to test this hypothesis.

Thyroglobulin-induced thyroiditis is a commonly used model for studies of HT [27] and can be established in susceptible non-obese diabetic (NOD) mice. These animals develop an HT-like illness that is characterized by monocytic infiltration of the thyroid gland and the presence of TPO-Ab and Tg-Ab autoantibodies, all of which are hallmarks of HT in humans [28]. These animals therefore represent an ideal euthyroid HT model system for studies of the contribution of microglia to the maintenance of neural circuits, and in this study they were therefore utilized to test our hypothesis.

## Methods

### Animals and study design

**Fig. 1:**
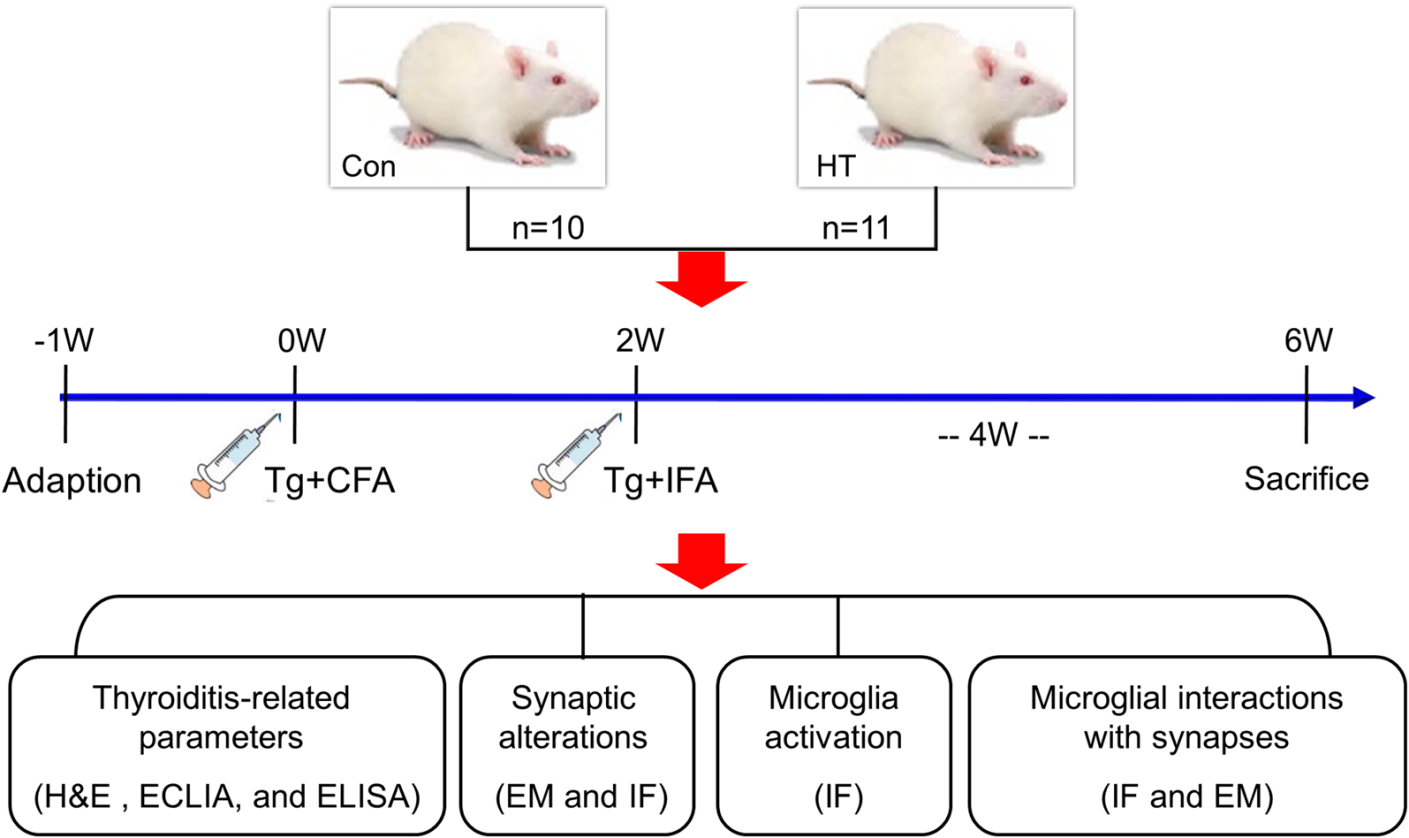
Experiment schedule. After one week of habituation, mice in the HT group were challenged twice with Tg emulsified in CFA followed by IFA boosts at 2-week intervals. Four weeks after the second challenge, the biochemical parameters of mice were evaluated

Twenty-one female NOD mice with approximately 8-week of age, weighing 21 ~ 24 g were obtained from Beijing HFK Bioscience (SCXK 2014-0004) and randomly divided into control (n = 10) or HT groups (n = 11). In this study, we only utilized female mice given that HT is more prevalent in women than in men [2], and given that female mice have primarily been used in prior preclinical studies of HT [29–31]. Mice were housed in groups of 3–4 at 23 ± 2 ℃, 55 ± 5%, humidity and a 12-h light/dark cycle with free access to standard food and water. After seven days of habituation, experimental HT was induced in mice based on methods described in our previous study [13]. Briefly, HT mice were immunized twice with porcine thyroglobulin (Tg; 25 µg) emulsified in complete or incomplete Freund's adjuvant (all were obtained from Sigma-Aldrich, USA) injected subcutaneously at 2-week intervals. Mice treated with phosphate buffered saline (PBS) instead of Tg at the same time served as controls. Four weeks after the last challenge, the behavioral and biochemical parameters of all animals were evaluated. The study design is shown in the flow-chart in Fig. 1. All procedures were carried out in accordance with the NIH Guide for the Care and Use of Laboratory Animals and approved by the Committee on the Ethics of Animal Experiments of Anhui Medical University (Anhui, China) (approval No. LLSC 20170169).

### Behavioral study

Spatial memory of the mice was assessed using the Morris water maze task. Mice were taken to the test room 60 min before the test. Behavioral procedures were conducted between 08:30 and 12:00 h in a dim and quiet room. The observers were blind to the experimental design. The protocol was conducted as we previously described [12].

### Tissue preparation

Mice were deeply anesthetized with pentobarbital (50 mg/kg, ip) and sacrificed by decapitation (09:30–12:00). Samples of the blood, brain, and thyroid were immediately obtained. Thyroid-related parameters were measured from blood samples. Histopathological evaluations were performed on the thyroid tissue. The brain samples were quickly collected for the subsequent experiments. Left frontal lobes were used for electron microscopy and electrochemiluminescence immunoassays and the right frontal lobes were used for RT-PCR, immunofluorescence, Nissl staining and immunohistochemistry (IHC). All mice were assigned to different experimental groups (n = 10–11 per group). The number of mice used for each data set is as follows: 4 left frontal lobes per group for EM; 6–7 left frontal lobes per group for ECLIA; 5 right frontal lobes per group for IF, IHC, and Nissl; 5–6 right frontal lobes per group for RT-PCR. All measurements were performed by a person who was unaware of the animal’s group.

### Electrochemiluminescence immunoassay (ECLIA)

ECLIA was used to analyze serum triiodothyronine (T3, Roche/Cobas/code no: 11731360 122), tetraiodothyronine (T4, Roche/Cobas/code no: 12017709 122), TPO-Ab (Roche/Cobas/code no: 11820818 191) and Tg-Ab (Roche/Cobas/code no: 04738578 191). A Cobas e411 immunoassay analyzer was used for all assessments (Roche, Germany) as previously described [32]. Frontal lobe T3 and T4 levels in mice were also assessed from homogenized tissue (PBS 1:9, weight to volume ratio). BCA assays (Cat#53225, Thermo Fisher Scientific) were used to measure protein concentrations in the supernatants. ECLIA was used for T3 and T4 levels in the frontal lobe which were normalized to total protein content.

### ELISA assays

ELISA assays were performed for the assessment of serum thyroid-stimulating hormone (TSH) levels (CEA463Mu, USCNLIFE, China). Data were expressed as picograms per milliliter of serum.

### Hematoxylin and eosin (H&E)

For pathological studies of the thyroid, tissues were paraffin embedded and H&E stained. Five non-contiguous sections (5 μm) from each thyroid were used for histopathology assessments. The thyroiditis classification standard was based on the percentage of thyroid infiltrated, as previously described [13]: 0 = absence of infiltrate; 1 = interstitial accumulation of inflammatory cells around one or two follicles; 2 = one or two foci of inflammatory cells reaching the size of a follicle; 3 = 10–40% inflammatory cells infiltration; 4 = greater than 40% inflammatory cells infiltration. The histological scores were evaluated and averaged by two investigators blind to experimental design.

### Electron microscopy (EM)

EM was performed to evaluate the alterations in synaptic density and morphology in mice. Briefly, areas in the frontal cortex were collected according to the mouse brain atlas [33]. Then the frontal lobes were trimmed into blocks of 1 mm3 with the aid of a cooled brain matrix (68707 and 68708; RWD Life Science Co., Shenzhen, China). EM procedures were performed as previously described [34]. Synapses were imaged at 20,000× and 50,000× magnification. We mainly photographed the region of the frontal lobe where the neuropil is located, which is composed mostly of dendrites, axons and synapses. Most cortical synapses (90–98%) are established in the neuropil [35]. Digital images at 20,000× (15 images per animal) were used to evaluate postsynaptic density (PSD) juxtaposed with presynaptic bouton-containing vesicles [36, 37]. For the analysis of frontal synaptic density, the number of synapses per unit volume of tissue (Nv) was counted on NIH ImageJ software (RRID: SCR_003070) and the numbers assessed using the stereological equation Nv = 8ENa/π2, as previously described [38, 39], where E designated the mean of the reciprocal values of the observed PSD lengths, and Na was synapse numbers per unit test area. In addition to synapse counting, we randomly selected 120 synapses from each group for analysis of synaptic ultrastructure. Only synapses with clear presynaptic and postsynaptic properties (Gray type I synapses) were assigned for the latter assay. We have added a series of EM images for two groups to provide more definite evidence in the supplementary material (Additional file 1: Fig. S1). For each condition, total presynaptic vesicles, active zone length, synaptic cleft width, PSD thickness, and synaptic curvature were measured according to previous criteria described by Jones [40].

In addition, the vicinity of microglia to neurons were assessed at 3000× to 20,000× magnification. Microglia were identified based on morphology assessments [26, 41] including irregular or oval nuclei with condensed chromatin (Fig. 10d) and/or cytoplasm with long endoplasmic reticulum (ER) cisternae (Fig. 9c). Neurons were identified by their larger cell bodies with organelle-rich cytoplasm (Fig. 10e). Synaptic innervation per neuron (80 neurons per group) was analyzed and expressed as the percentage area of the neuronal cell body covered by presynaptic terminals, as previously described [26].

### Immunofluorescence (IF)

For IF analysis, 3 coronal sections (1/5 serial sections) were assessed. Sections were probed with mouse anti-synaptophysin (anti-SYN, 1:100, Cat#MAB5258, Millipore, RRID:AB_95187), mouse anti-postsynaptic density protein 95 (anti-PSD95, 1:200, Cat#MA1-045, Thermo Fisher Scientific, RRID:AB_325399), and rabbit anti-Iba1 (1:100, Cat#019-19741, Wako, RRID: AB_839504) primary antibodies. The appropriate fluorophore-conjugated secondary antibodies were purchased from Servicebio Inc., followed by DAPI staining (Cat#G1012, Servicebio, China) staining. Samples were scanned on a OLYMPUS IX73 fluorescence microscope. SYN- and PSD95-staining was observed at the frontal lobe areas at 400× magnification. Iba1- and Iba1/PSD95- stained images were captured at 200× and 730× magnification, respectively.

For quantification of frontal synapse, the sections were stained for pre- and postsynaptic- markers, SYN and PSD95, respectively. According to a previously reported method [42], ten magnified images in each section were captured randomly in the frontal lobe for detection of the number of SYN + and PSD95 + puncta with ImageJ. The number of SYN- and PSD95-labeled puncta were normalized with data from the controls to determine the relative synaptic number.

For analysis of microglial activation, microglia were visualized using antibodies against Iba-1, an effective marker for activated microglia. According to previously used method [43, 44], microglial activation was identified by quantifying the number and phenotype of Iba1-labeled cells. The morphological phenotype of Iba1-labeled microglia was divided into three different subtypes: ramified (surveying) (RAM), intermediate (INTER), and round/amoeboid (phagocytic) (R/A). Values were obtained from five mice for each group, three slices from each animal and three random images from each slice.

To quantify the engulfment of synapses by the microglia, Iba-1 and PSD95 co-staining was performed as previously described [37, 45]. The frontal lobe was imaged by a zeiss 800 confocal microscope system equipped with a × 63 oil immersion objective (ZEISS, Germany) by using identical light intensity and exposure settings in stacks (z-step 0.1 μm). The images of contact between microglia and postsynaptic structures in identical × 60 image stacks from sections double-labeled for Iba1 and PSD95 were processed by the ZEN blue software (ZEISS, Germany). Forty microglia per animal were randomly captured in the frontal lobe for detection of the number of PSD95-labeled puncta that localized inside Iba1-labeled cells.

### Immunohistochemistry (IHC)

For IHC, 3 coronal sections were assessed per animal using serial Sects. (1/5). Sections were dewaxed, hydrated, and antigen retrieval was performed. Endogenous peroxidase was quenched and sections were blocked in serum. Sections were labeled with the appropriate primary and secondary antibodies and (DAB) and hematoxylin stained. The primary antibody was anti-Iba1 (1:400, Cat#019-19,741, Wako), which was chosen for immunostaining for its specificity to microglia and labeling of the entire microglial body, including processes. The slides were viewed randomly in the frontal lobe areas at a 400× magnification with a Nikon 80i microscope (Japan). Meanwhile, low IHC images to specify the area within the frontal lobe were provied in Additional file 2: Fig. S2. For analysis of microglial-neuronal interactions, the number of microglia-neuron overlaps in the frontal lobe were counted with Image J. Neurons were stained with hematoxylin [46]. The overlap of microglia-neurons was identified by the appearance of microglial cell body-neuronal cell body contacts as described by Bolton et al. [47].

### Nissl staining

Nissl staining was used to estimate neuronal numbers in the frontal lobe regions of mice. Five paraffin-embedded sections (1/5 serial sections) per animal were stained with 1% toluidine blue solution (Cat#G1032, Servicebio, China) for 25 min at 37 ℃, dehydrated through an gradient ethanol, and then mounted with Permount. Tissues were scanned on a light microscope (Eclipse 80i, Nikon, Japan). In each section, ten non-overlapping fields were randomly captured at magnification 400×. Neuronal numbers were identified by pale-stained cytoplasm rich in the Nissl substance and large nuclei containing a prominent nucleolus [48, 49], were calculated and averaged by two observers in a blind fashion using ImageJ.

### RT-PCR

Frontal lobe RNA was extracted using TRIzol (Cat#15596018, Invitrogen). RNA samples were reverse transcribed into cDNA using a Revert Aid™ First-Strand cDNA Synthesis Kit (Cat#K1622, Thermo Fisher Scientific). PCR primers were as follows: Synaptophysin: forward primer (FP) -AGA CAT GGA CGT GGT GAA TCA, reverse primer (RP)- ACT CTC CGT CTT GTT GGC AC; PSD95: FP-TCC GGG AGG TGA CCC ATTC, RP-TTT CCG GCG CAT GAC GTAG; 18s: FP-GTA ACC CGT TGA ACC CCA TT, RP-CCA TCC AAT CGG TAG TAG CG. PCRs were performed in duplicate on a Light Cycler® 480 (Roche). The 18s gene was used as an internal reference. Relative mRNA levels were analyzed using the 2-ΔΔCt method.

### Statistical analysis

Data were analyzed using GraphPad Prism 7.0 (SCR_002798, GraphPad Software, USA). A Kolmogorov–Smirnov test of normality was performed for all variables. When parametric analysis was possible, the unpaired Student’s t-test was performed for parameter comparisons between the groups. In cases when parametric analysis was not possible, the Mann–Whitney U test was applied to assess the significance of differences between the results. Data are reported as means ± SEM. A P-value < 0.05 was considered statistically significant.

## Results

### Euthyroid HT models

**Fig. 2:**
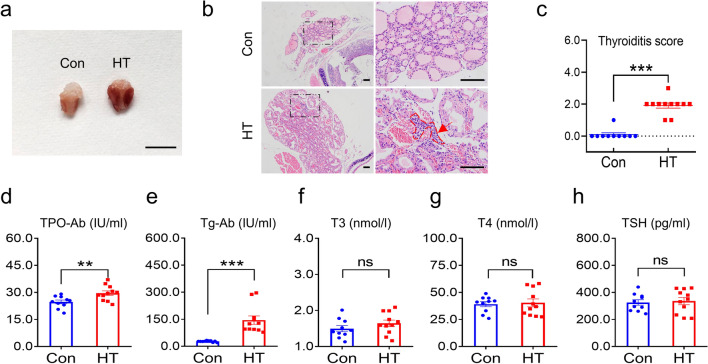
Mouse models of euthyroid HT. **a** Gross inspection of thyroids from HT and Con Mice. Note the enlarged thyroids in HT mice. **b** Representative thyroid sections stained with H & E showing monocyte infiltration between the groups (arrow). The control mice had intact thyroid follicles with an even distribution, and monocyte infiltration was hardly found in thyroid tissues. In contrast, HT mice displayed disorderly and destroyed thyroid follicles, and monocyte infiltration was evident in thyroid tissues. **c** Quantitation of monocyte infiltration. **d–h** Serum levels of the indicated thyroid-related parameters. Data are the mean ± SEM (Con group, n = 10 mice; HT group, n = 11 mice); ns, no significant difference; *p < 0.01, **p < 0.01, and ***p < 0.001. Scale bars: = 100 μm

A flow chart of the study design is shown in Fig. 1. We assessed the euthyroid HT model via histological and serological examinations. The mice developed enlarged thyroids after Tg immunization (Fig. 2a). Histological observations revealed inflammatory infiltration in the thyroids of HT mice, whilst minimal monocyte infiltration was evident in the control group (Fig. 2b). HT mice showed more severe HT following quantitative analyses (Fig. 2c). Serum TPO-Ab and Tg-Ab in the HT mice increased compared to controls (Fig. 2d, e). No significant alterations in serum T3, T4 or TSH levels were observed (Fig. 2f-h). This confirmed the successful establishment of the euthyroid HT model.

### Euthyroid HT induces learning and memory dysfunction

**Fig. 3:**
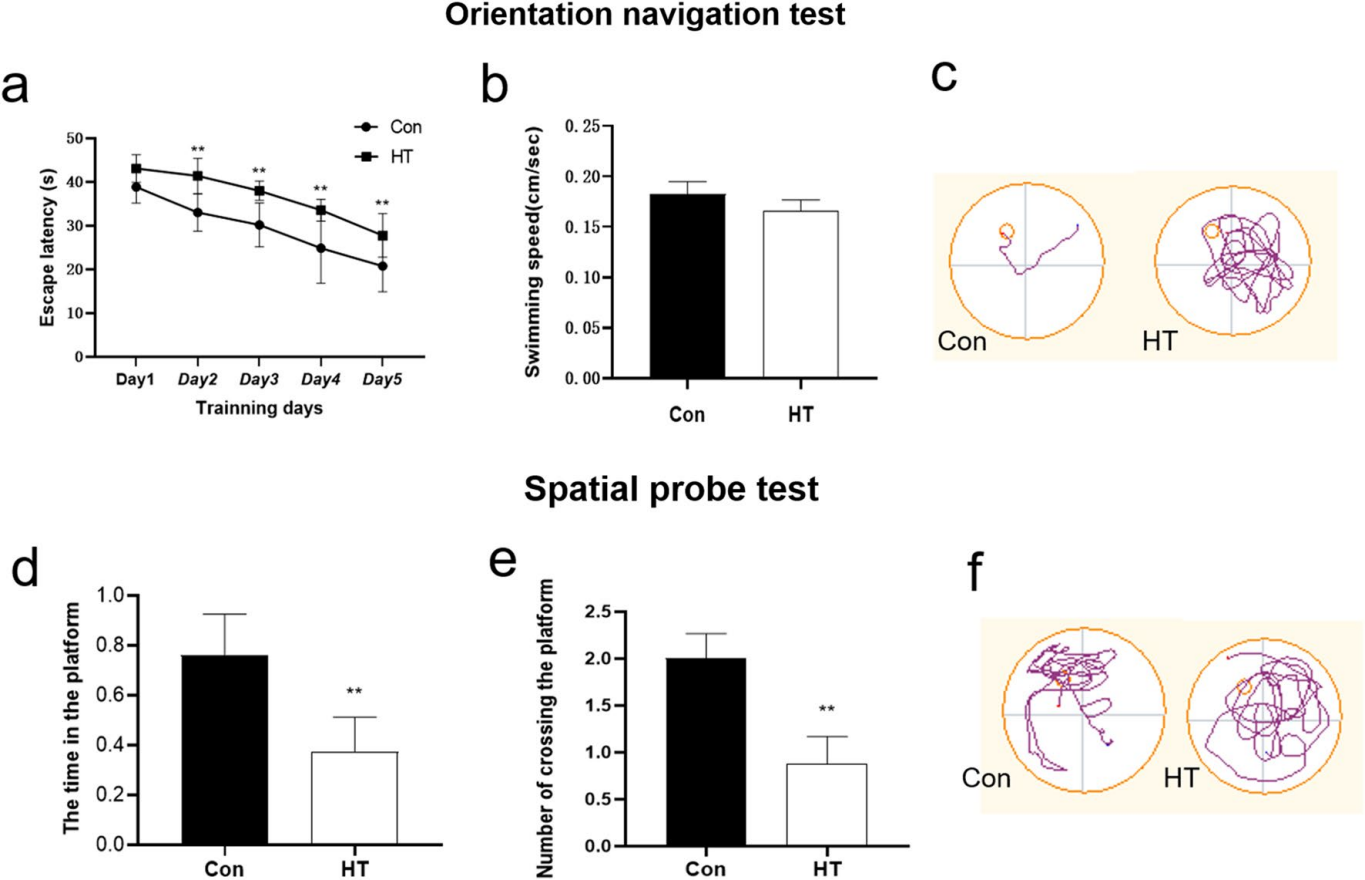
Euthyroid HT induces poor performance in Morris water maze in mice. **a** The average escape latency period for the mice to find the underwater platforms. Following a 5-day training session, the latency to find the escape platform was progressively shortened for both Con and HT mice, but the HT mice took a significantly longer time to find the escape platform compared to the control group at the 2nd to 5th training days. **b** No significant difference in swimming speed was seen between the two groups. **c** The representative path tracking of the Con and HT mice in orientation navigation tests. **d**, **e** The time spent on the platform and the number of crossing the platform in spatial probe tests. **f** The representative path of the Con and HT mice in spatial probe tests. Data are presented as the mean ± SEM; *p < 0.05, and **p < 0.01, vs. Con

The escape latency time of two training groups became shorter as training days increased. Comparing with the Con group, mice in the HT group took a significantly longer time to find the escape platform (Fig. 3a). Meanwhile, there was no statistically significant difference between the Con group and the HT group in swimming speed (Fig. 3b), and in the spatial probe test, the time spent on the platform and the number of crossing the platform decreased in the HT group (Fig. 3d, e). The typical path tracking of each group was shown in Fig. 3c and 3f. These results suggest that the mice in the HT group have learning and memory dysfunction.

### Frontal lobe levels of T3 and T4

**Fig. 4:**
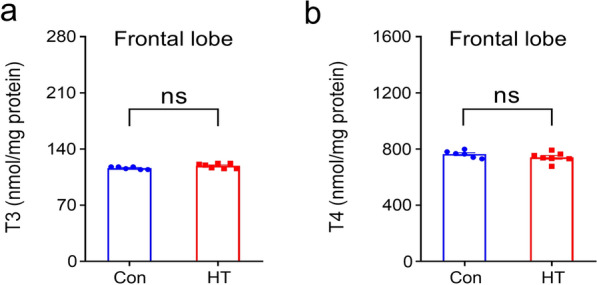
Frontal lobe levels of T3 and T4. Thyroid hormones in mouse brain homogenate supernatants were measured by ECLIA. **a** Frontal lobe T3; **b** Frontal lobe T4. Data are the mean ± SEM (Con group, n = 6 Con group, n = 10 mice; HT group, n = 7 Con group, n = 10 mice); *P*-values as in Fig. 2

Previous studies found that local deficiency of thyroid hormones was seen prior to the decrement in plasma [50]. Accordingly, we evaluated frontal lobe T3 and T4 levels in all animals. As shown in Fig. 4, no differences were observed in T3 or T4 concentrations in the frontal lobe.

### Euthyroid HT induces ultrastructure changes in frontal synapses

**Fig. 5:**
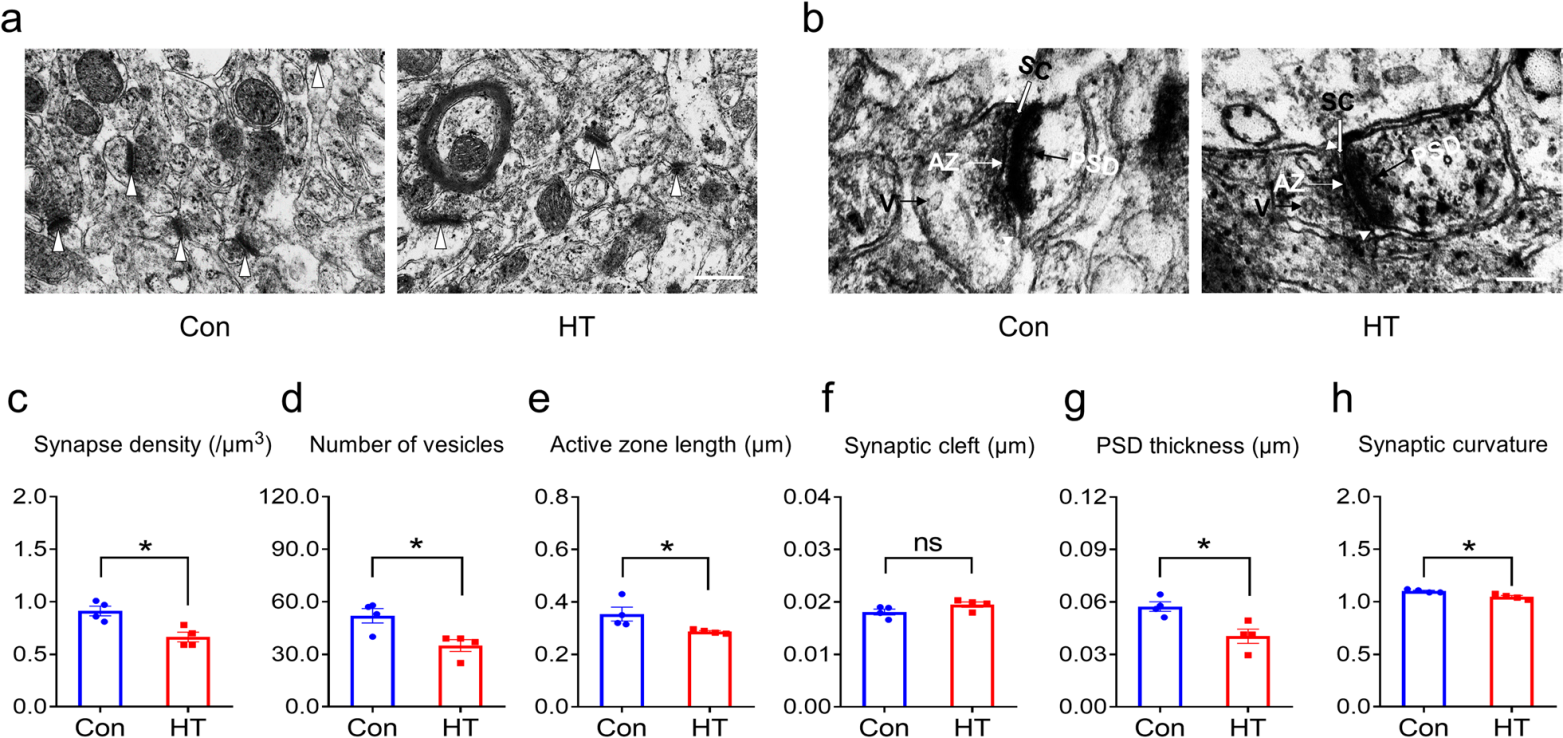
Euthyroid HT induces ultrastructure changes in frontal synapses. **a** Representative EM images of synapses (arrowheads). **b** Representative EM images of synaptic interface; V represents synaptic vesicles; the synaptic active zone is indicated by white arrows; SC represents the synaptic cleft; PSD is indicated by black arrowheads. **c–h** Quantitation of the synaptic interface, including synapse density (**c**), number of vesicles (**d**), active zone length (**e**), synaptic cleft (**f**), PSD thickness (**g**), and synaptic curvature (**h**). Data are the mean ± SEM (n = 4 mice/group); Scale bars: a = 500 nm; b = 200 nm

To examine the effect of HT on frontal synapses, synapse morphometry was examined using EM. HT and control groups showed significant differences in synaptic density and interface parameters (Fig. 5a, b). Quantitatively, the ultrastructure of the frontal lobe neurons revealed lower synaptic density (Fig. 5c), fewer presynaptic vesicles (Fig. 5d), shorter active zone length (Fig. 5e), thinner postsynaptic densities (Fig. 5g), and decreased synaptic curvature (Fig. 5h) in the HT mice compared with those in the Con mice. The other synaptic interface parameters, such as synaptic cleft, did not show differences between groups (Fig. 5f). The morphological data suggested that euthyroid HT could induced synaptic loss and impaired synaptic ultrastructure in the frontal lobe regions of mice.

### Euthyroid HT induces synaptic loss in the frontal lobe

**Fig. 6:**
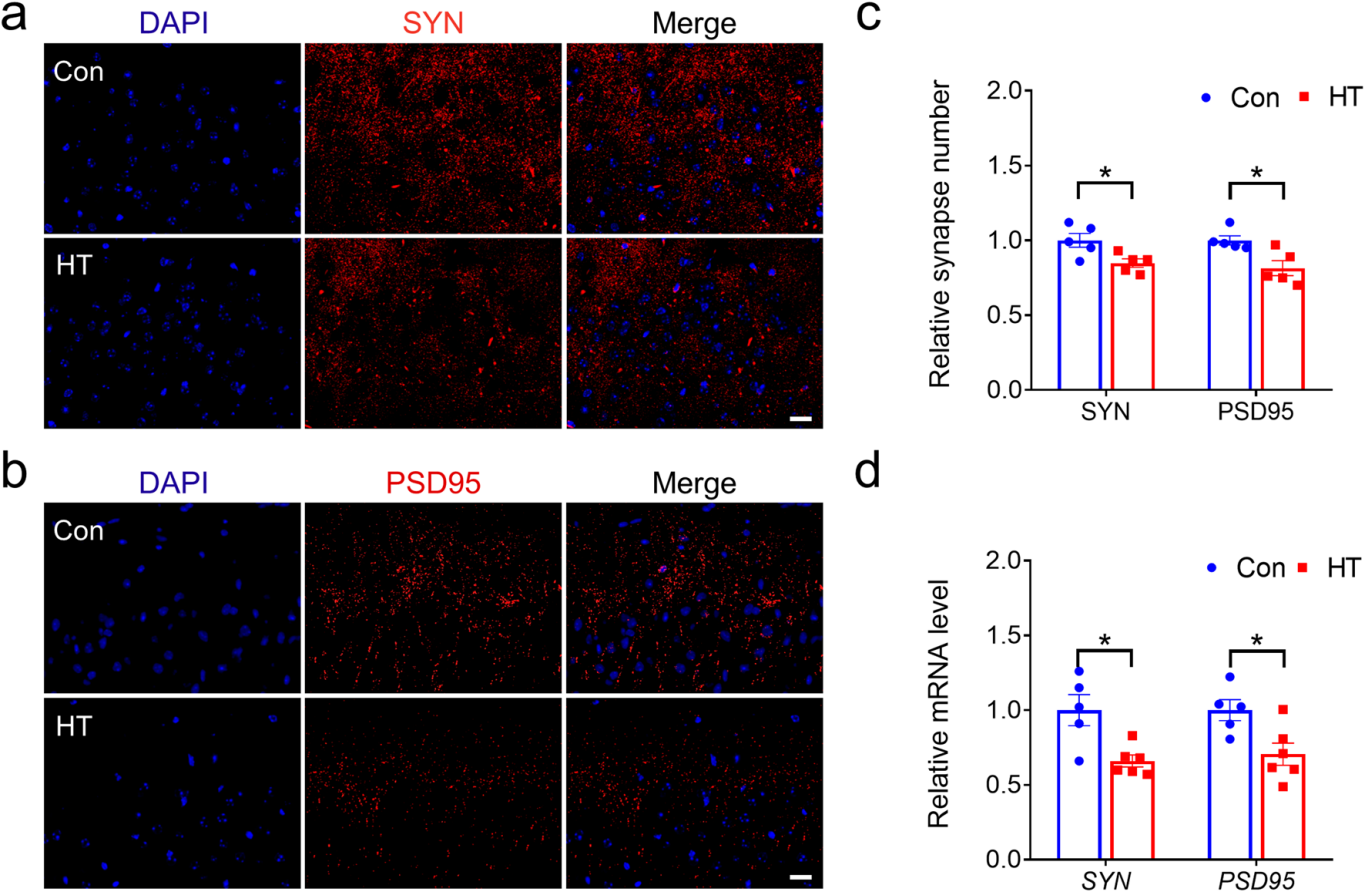
Euthyroid HT induces frontal lobe synaptic loss. **a**, **b** Representative IF images of SYN-labeled puncta (**a**) and PSD95-labeled puncta (**b**). **c** Quantitative decrease in the number of SYN and PSD95-labeled puncta in the frontal lobe of HT mice (n = 5 mice /group). **d** *SYN* and *PSD95* mRNA expression (Con group, n = 5 mice; HT group, n = 6 mice). Data are the mean ± SEM; Scale bars = 20 μm

To characterize HT-induced synaptic loss, we performed immunostaining with antibodies against the presynaptic maker SYN and the postsynaptic maker PSD95 to determine if HT decreases synaptic numbers in the euthyroid state. SYN and PSD95 showed a punctate staining pattern (Fig. 6a, b). According to previous studies [42], we quantified the density of SYN- and PSD95- labeled puncta in the frontal lobes of mouse models. The frontal lobe of the HT group showed fewer presynaptic and postsynaptic puncta compared to control groups (Fig. 6c), indicating that euthyroid HT decreases the number of synapses in the frontal lobes of mice. Subsequent quantitative analysis of SYN and PSD95 mRNA expression confirmed these findings (Fig. 6d).

### Neuronal loss is not observed in the frontal lobes of HT mice

**Fig. 7:**
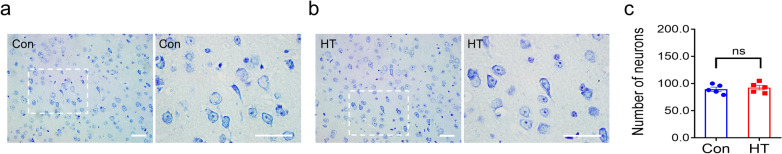
Neuron loss does not occur in the frontal lobes of HT mice. IF images of Nissl-labeled neurons in Con (**a**) and HT mice (**b**). Each right panel (×1000) depicts a magnified image of the boxed in the left panel (×400). Neuron numbers were comparable between groups (**c**). Data are the mean ± SEM (n = 5 mice /group); Scale bars = 20 μm

Nissl staining of the frontal lobes showed no significant differences in the number of Nissl-labeled neurons between the groups (Fig. 7).

### Microglia activation occurs in the frontal lobes of HT mice

**Fig. 8:**
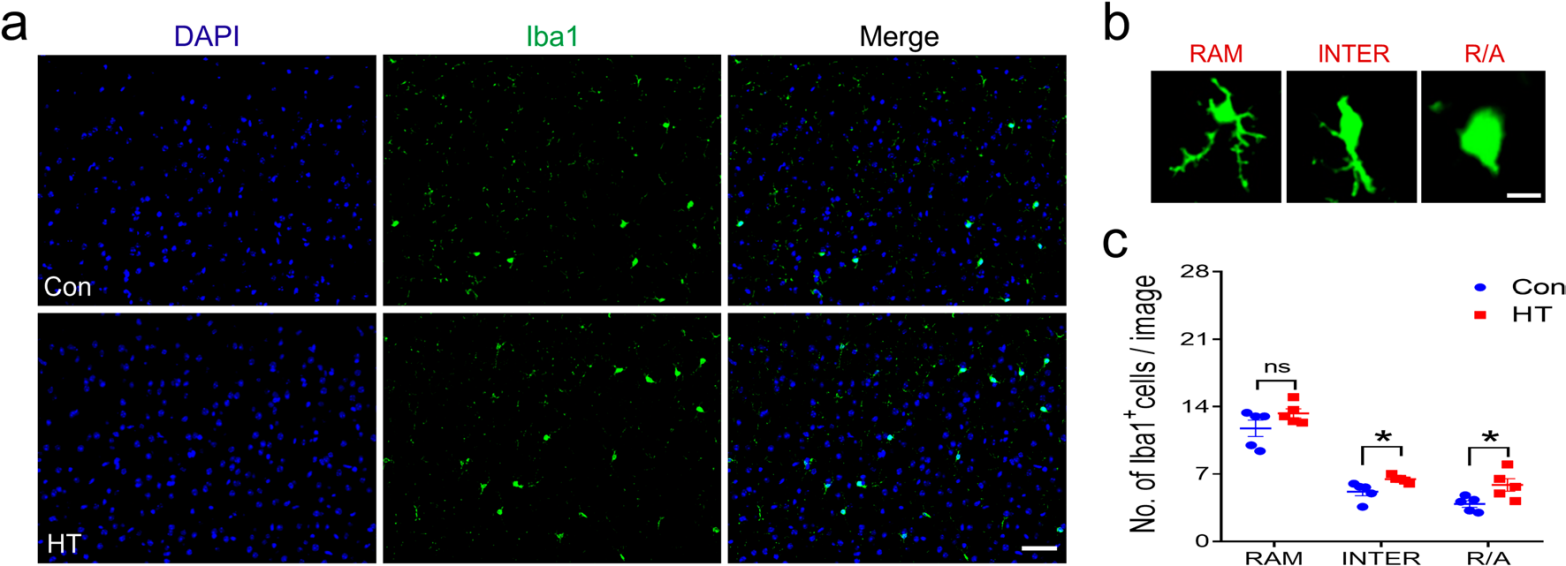
Frontal lobe microglia activation in HT mice. **a** Images of Iba1^+^ microglia (green). Blue: DAPI. **b** Microglial morphology including RAM, INTER and R/A Iba1^+^ cells. **c** Number of Iba1^+^ cells with each morphology (n = 5 mice /group). Data are the mean ± SEM; Scale bars: a = 50 μm; b = 10 μm

We next analyzed the morphology of microglial cells due to their close correlation with activation states [51]. We quantified the phenotype and number of Iba1-labeled cells (RAM, INTER and R/A) using IF as a surrogate marker of microglial activation (Fig. 8a, b). We examined the microglial response in the frontal lobes of HT mice and observed a significantly higher percentage of INTER and R/A Iba1 + cells, suggesting an activated microglial phenotype in HT compared to control mice (Fig. 8c).

### Euthyroid HT increases the microglial engulfment of synaptic elements in the frontal lobe

**Fig. 9:**
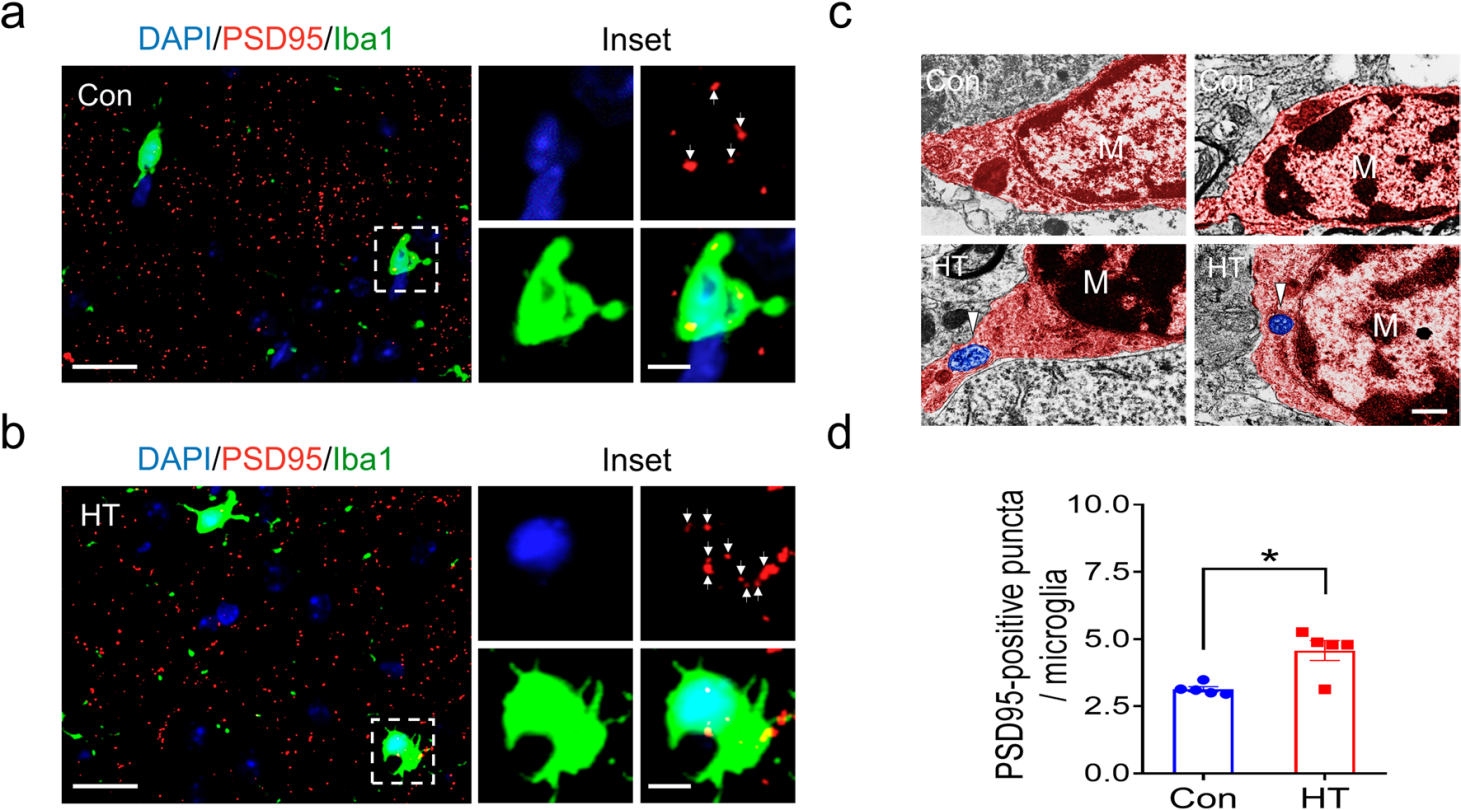
Euthyroid HT increases microglial engulfment of synaptic elements in the frontal lobe. **a**, **b** Representative images showing immunofluorescence labeling for the microglial marker Iba1 (green) and the synaptic marker PSD95 (red) in the frontal lobe of control (**a**) and HT (**b**) mice. The nuclei was stained with DAPI (blue). Insets shows localization of PSD95-labeled puncta (arrow) in microglia at higher magnification. **c** Representative EM images of synaptic elements (arrowhead) engulfed by microglia (M; blue) in HT mice. **d** PSD95-labeled puncta per microglia. Data are the mean ± SEM (n = 5 mice /group); Scale bars: **a** and **b** = 20 μm; insets = 5 μm; **a** = 500 nm

The activation of microglia towards an amoeboid morphology is associated with increased phagocytosis [45]. We therefore determined whether euthyroid HT promoted microglial phagocytosis of the synaptic elements in the frontal lobe using immunofluorescence labeling. As shown in Fig. 9a, b, PSD95-labeled synapses were engulfed by Iba1-labeled microglia in the frontal lobe of control and HT mice. Quantitative analysis indicated that the number of PSD95-labeled puncta engulfed by microglia were significantly higher in HT compared to controls (Fig. 9d). EM revealed that the synaptic structures mainly membrane-bound compartments containing synaptic vesicles were visible within the microglia of HT mice (Fig. 9c) but were rarely observed in control mice, so it was not possible to conduct quantitative analysis.

### Euthyroid HT increases microglial attachment to neurons and decreases the number of presynaptic terminals covering the neurons in the frontal lobe

**Fig. 10:**
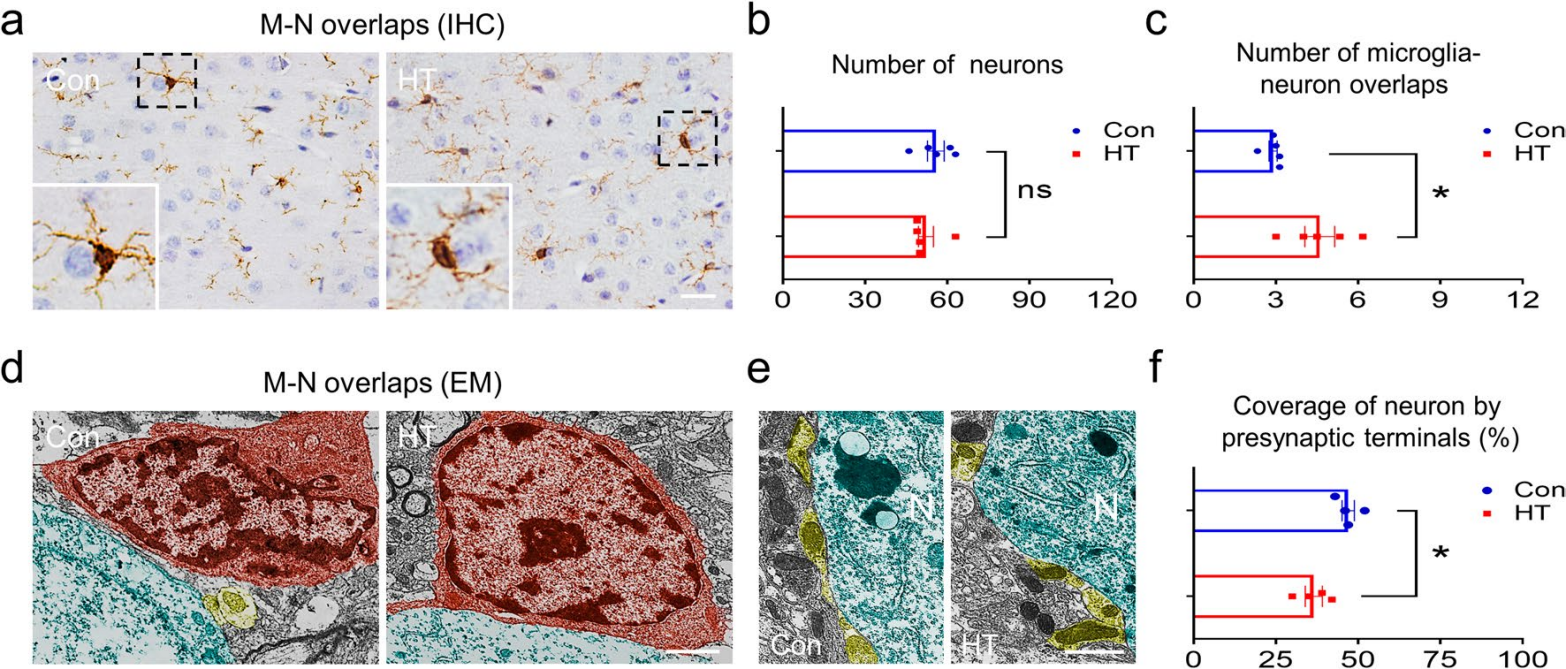
Euthyroid HT increases frontal lobe microglial attachment to neurons and decreases presynaptic terminals covering neurons. **a** Representative images of Ibal-labeled microglia and hematoxylin-stained neurons in mice (n = 5/group). Magnified images of the boxed areas are shown. **b** Neuronal quantification. (**c** Number of microglia-neuronal overlaps. **d** Representative EM images of microglia-neuron interactions, indicating an abnormal apposition of microglia with neurons in euthyroid HT mice using EM that was not quantified. Surface areas of neurons (green) of HT mice occupied by microglia (red) were bare of synaptic endings (yellow), whilst neurons (green) juxtaposed with microglia (red) in controls had abundant synaptic contacts (yellow). **e** EM images of presynaptic terminals (yellow) covering neurons. **f** % of the neuronal cell surface covered by presynaptic terminals (yellow) (n = 4 mice /group). Data are the mean ± SEM; Scale bars: a = 20 μm; d = 2 μm; e = 1 μm

Reactive microglia can strip presynaptic terminals from neuronal perikarya in some pathological conditions [24, 52], therefore, we extended the work to study the influence of microglial morphological alterations on neuronal synaptic networks. We first observed the influence using immunohistochemistry and quantified the number of microglial-neuronal overlaps in the frontal lobe using Image J. As shown in Fig. 10a, a number of cell body-cell body overlaps were viewed between Ibal-labeled microglia and hematoxylin-stained neurons. Such interactions were quantified to examine whether they were influenced by euthyroid HT. Mice in the HT group contained significantly more overlaps compared to mice in the Con group (Fig. 10c), suggesting that microglia displace synaptic inputs in the neurons [53]. There was no significant overall difference in numbers of neurons between groups (Fig. 10b). Interestingly, we also observed an abnormal apposition of microglia with neurons in euthyroid HT mice using EM that was not quantified (Fig. 10d). To make a clear case for interaction between microglia and synapses and possible correlation, EM was used to investigate the consequences of the interactions. The surface areas of the neurons of HT mice fully occupied by microglia were bare of synaptic endings, whilst the neurons of control mice were covered by synaptic contacts. In view of this, we analyzed the percentage of the neuronal cell surface occupied by presynaptic terminals between groups as previously described [26]. As shown in Fig. 10e, f, HT mice displayed a significant reduction in the percentage of presynaptic terminals covering the neurons. Briefly, these morphological data provided evidence that microglia migrate to and strip synapses from the neuronal soma in euthyroid HT.

## Discussion

This study performed a behavioral experiment and comprehensive morphological assessment and quantitative synaptic ultrastructural analysis to determine pre- and postsynaptic protein expression in euthyroid HT mice. Morris water maze experiment is a classic method to evaluate the ability of spatial learning and memory by forcing experimental animals to look for the underwater hiding platform [25]. In the present study, mice in the HT group took a significantly longer time to find the hidden platform, had a reduced time spent in the target platform, and a decreased number of crossing the platform compared to mice in the Con group. This confirmed that HT itself can induce spatial learning and memory impairment regardless of thyroid functions in mice, which is in accordance with our previous study. Concurrently, our results provide initial evidence that euthyroid HT induces synaptic loss and alters the synaptic ultrastructure of mice. Interestingly, we observed microglia activation and enhanced phagocytosis of synaptic materials, in addition to an abnormal apposition of microglia to neurons in euthyroid HT mice, suggesting a mechanism of microglia-mediated synaptic loss, which may underlie the deleterious effects of HT on spatial learning and memory.

Synapses act as interfaces in which networks of neurons interact during physiological brain function [54]. We found that euthyroid HT produced a reduction in synaptic density, and reduced the number of vesicles, active zone length, PSD thickness, and synaptic curvature in the frontal lobe at the ultrastructural level. The changes indicated that euthyroid HT induced synaptic loss and impaired synaptic structures in mice. Active regions and PSD are key elements of complex synaptic signals that mediate synaptic plasticity [55]. Curvature can be observed at the interface of pre- and post-synaptic areas, designed to enhance surface area, ensuring direct delivery of the delivered transmitters as opposed to diffusion across the peripheral space. This enhances the efficacy of neurotransmission [56]. Consistent with these data, the quantification of SYN and PSD95 revealed synaptic loss in the frontal lobes of HT mice. Studies have confirmed that SYN, PSD95 and other synaptic proteins regulated the release of neurotransmitters and dictated the plasticity of synapses [20]. Altering these parameters contributes to altered plasticity at the structural and functional level, impairing the efficiency of synaptic transmission between neurons in HT, leading to neuropsychological HT alterations.

How HT leads to a loss of synapses in the euthyroid remains unclear. Thyroid dysfunction can induce neuronal deficits, including synaptic alterations [57, 58]. Our previous studies have highlighted that experimental hypothyroidism induces synaptic deficit in the hippocampus [59] and prefrontal lobe [34]. Interestingly, local deficiency (e.g., in the hippocampus) of thyroid hormone was observed prior to the decrement in plasma in an animal model of subclinical hypothyroidism [50]. In this study, T3 and T4 levels were detected in the frontal lobe but no differences were found between groups, indicating that the synapse loss in the euthyroid HT mice had nothing to do with differences in local thyroid hormones. On the other hand, thus far, synapse loss has been proposed to occur via neuron-autonomous mechanisms (for example, apoptosis) [60, 61] or synaptic stripping mediated by the glia [37, 62]. Evidence for either scenario or a combination of both exists. As an exemplar, Albert et al. showed a loss of dentate afferent synapses and from ultrastructural data highlighting autonomous and glia-mediated degradation of synapses in chronic multiple sclerosis (MS) [63]. In this study, synapse loss was unlikely to be due to local neuronal cell loss in HT mice, because neuron numbers were within the normal range for this brain area. This was consistent with our previous findings showing a lack of neuronal apoptosis or ultrastructural damage in the frontal lobe of euthyroid HT mice [13].

Synaptic loss has also been reported in other animal models of autoimmune illness, such as systemic lupus erythematosus (SLE) [64] and MS [63]. The close-association of synapses and microglia may contribute to synapse loss [64, 65] a factor that has been poorly investigated in HT mice. During activation, microglia undergo clear morphological changes. Our findings indicated that the number of round/amoeboid Iba1-labeled cells, a reactive microglia phenotype [44] significantly increased in the frontal lobe of HT mice. Microglial activation enhances phagocytosis. An intriguing hypothesis is therefore that HT enhances microglial phagocytosis of synaptic elements, contributing to synapse loss in euthyroid HT. Consistent with this hypothesis, increased PSD95 engulfment occurred in HT mice. We interpreted the presence of PSD95-labeled puncta in microglia as a consequence of active phagocytosis of synapses. Indeed, EM observations showed that membrane-bound compartments containing synaptic vesicles were visible within the microglia of HT mice. Similar EM observations have been reported in lupus-prone mice, in which microglial cells are reactive and engulf synaptic material, suggesting microglia-dependent synaptic loss [64].

The activation of microglia is accompanied by chemotactic responses and migration towards areas of neuronal damage [66, 67]. We thus explored the physical associations between microglia and neurons in the frontal lobe. Microglial attachment to neuronal perikarya increased in HT mice. In addition, at the ultrastructural level, microglial cells directly contacted neuronal cell bodies lacking synapses, a phenomenon rarely observed in control mice. An abnormal association of microglia and neurons has been reported in MS [68] and Alzheimer’s disease [69]. A consequence of close apposition between microglial cells and neurons is the interruption of synaptic contacts [25, 26, 41], termed synaptic stripping [67], also reported in animal models of cortical inflammation [26]. Morphological studies [21, 70] have revealed direct contact between microglia and synaptic elements supporting the possibility that microglia strip synaptic elements. Consistent with these data, we provide preliminary morphological evidence that microglia in euthyroid HT have closer interactions with synapses than that in control.

Synaptic elimination might be mediated by microglial phagocytosis as a consequence of direct synaptic removal [71]. However, microglial cells secrete synaptotoxic factors, including TNF-α and IL-1β [72]. In vitro studies revealed that conditioned medium from reactive microglia led to a loss of synaptophysin in primary neuronal cultures [73]. In addition, some soluble neurotrophic factors [23], which were secreted by microglia, could be crucial for synapse maintenance, therefore, dysfunction in homeostatic microglial activity may result in a lack of such support, thereby contributing to synaptic loss. Our previous work demonstrated that euthyroid HT increased the expression of TNF-α and IL-1β in the frontal lobes of mice [13]. Thus, synaptic loss following euthyroid HT may be, at least partially, attributed to activated microglia. This suggests that the inhibition of microglial activation offers neuroprotection during euthyroid HT.

Our study also has some limitations. We assessed the role of microglia in synaptic remodeling in the frontal lobe. However, the synaptic loss that accompanies synaptic ultrastructure damage in this brain area may not be only attributable to reactive microglia. Astrocytes are the most numerous cell in the central nervous system that provide trophic support for neurons, promote formation and function of synapses, prune synapses by phagocytosis, and fulfil a range of other homeostatic maintenance functions [74–77]. Previous studies found that neuroinflammation and ischaemia induced two different types of reactive astrocytes that they termed A1 and A2, respectively. A1 astrocytes highly upregulate many classical complement cascade genes previously shown to be destructive to synapses, so they postulated that A1 astrocytes might be harmful. By contrast, A2 astrocytes upregulated many neurotrophic factors, and they therefore postulated that A2 astrocytes were protective. Recent data revealed that A1 neurotoxic astrocytes were induced by activated microglia [78]. In fact, our previous study revealed increased activation of astrocytes using the same HT model, in which we identified a greater number of activated cells and a greater area of GFAP expression in HT mice than in controls [13]. Astrocytes lead to synaptic loss both directly (through recognition of a phagocytosis signal from synaptic components) and/or indirectly (by inducing the deposition of certain complement proteins at the synapses, which are subsequently eliminated by microglia) [79]. Further studies are now required to determine whether astrocytes contribute to synaptic loss in euthyroid HT. But this is not the topic of our present study. Furthermore, the study of the interactions between microglia and synapses in HT should extend to other brain region such as the hippocampus, which is a brain region essential to spatial learning and memory. We hope to continue to improve these limitations in our subsequent studies.

## Conclusions

In summary, our data show initial evidence that HT induces synaptic loss in the euthyroid state with deficits might be attributable to activated microglia, which may be the mechanism underlying the spatial learning and memory impairment in HT. This offers important clues into how activated microglia affect neural circuits following HT and provides a new perspective to neuroprotection in euthyroid HT.

## Supplementary Information

**Additional file 1: Fig. S1.** A series of EM images for two groups at low (× 4000) and high (×10,000) magnification. Synapses are indicated by red arrowheads.

**Additional file 2: Fig. S2.** IHC images for two groups at low (×100) and high (×400) magnification. Each right panel (× 400) depicts a magnified image of the boxed in the left panel (×100).

## Data Availability

The datasets used and/or analyzed during the current study are available from the corresponding author on reasonable request.
