# Supplementary figures and images for: Synaptic loss in a mouse model of euthyroid Hashimoto’s thyroiditis: possible involvement of the microglia
Source: BMC Neurosci. 2022 Apr 25;23:25. doi: 10.1186/s12868-022-00710-2 (PMC9036731; doi:10.1186/s12868-022-00710-2)

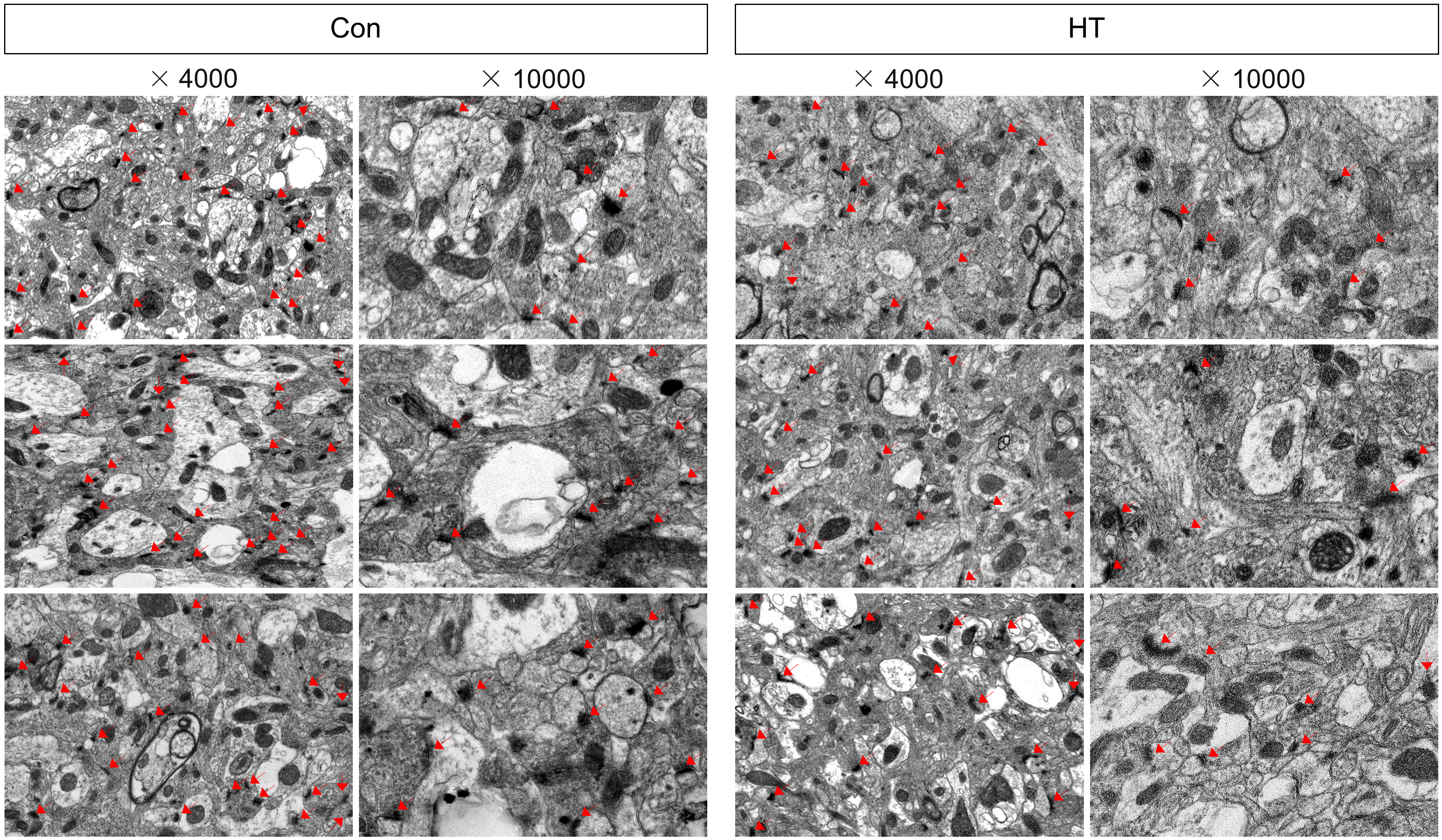

Supplement: Supplementary file 1 — Additional file 1: Fig. S1. A series of EM images for two groups at low (× 4000) and high (×10,000) magnification. Synapses are indicated by red arrowheads. [file 12868_2022_710_MOESM1_ESM.tif]

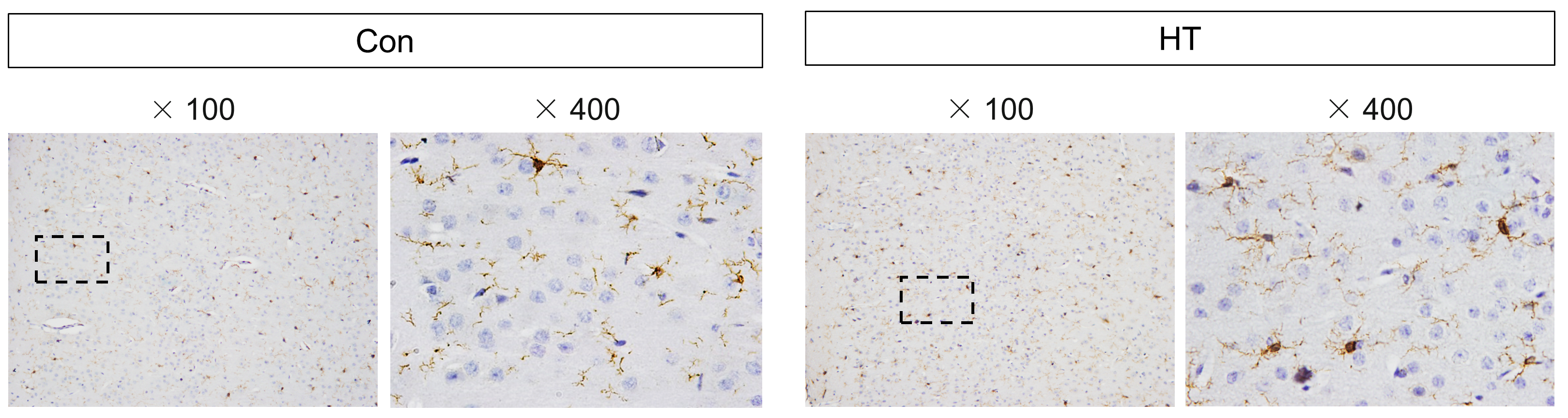

Supplement: Supplementary file 2 — Additional file 2: Fig. S2. IHC images for two groups at low (×100) and high (×400) magnification. Each right panel (× 400) depicts a magnified image of the boxed in the left panel (×100). [file 12868_2022_710_MOESM2_ESM.tif]
